# Body of Proof: Biomonitoring Data Reveal Widespread Bisphenol A Exposures

**DOI:** 10.1289/ehp.118-a353a

**Published:** 2010-08

**Authors:** Kellyn S. Betts

**Affiliations:** **Kellyn S. Betts** has written about environmental contaminants, hazards, and technology for solving environmental problems for publications including *EHP* and *Environmental Science & Technology* for more than a dozen years

A review of more than 80 biomonitoring studies from nine nations suggests exposure to bisphenol A (BPA) is ubiquitous in people throughout the world **[*EHP*** **118(8):1055–1070; Vandenberg et al.]**. Moreover, in samples of blood serum, median levels of unconjugated (biologically active) BPA were higher than levels predicted by toxicokinetic models that form the basis of U.S. regulations for the compound, reaching the range that has been shown to cause adverse effects in animals.

More than 8 billion pounds of BPA are produced each year, making it one of the world’s most heavily used chemicals. BPA is used in baby bottles, drinking bottles, food storage containers, polyvinyl chloride, stretch films, paper, cardboard, medical equipment, and the epoxy resins lining most metallic food and beverage cans. BPA has estrogenic properties, and animal studies have linked low-level exposure to altered development of the male and female reproductive tract and brain as well as cancers of the mammary gland and prostate.

The authors analyzed 24 biomonitoring studies involving blood serum samples from healthy adults, adults with certain diseases, pregnant women, and fetuses or fetal tissues. Overall, these studies indicate exposure to unconjugated BPA is in the range of 0.5–10 ng/mL, with most studies suggesting an average exposure of 1–3 ng/mL. The latter concentrations are higher than those shown to cause effects in human and animal cell cultures.

The only data on BPA levels in children after birth is from studies of urine samples, most of which measured total (conjugated and unconjugated) BPA, but some of which measured unconjugated BPA separately from conjugated (inactive) BPA. The Centers for Disease Control and Prevention measured total BPA in urine samples from 314 children aged 6–11 years and 715 adolescents aged 12–19. Compared with adults, the younger children’s levels were highest, and adolescents were in-between. Other smaller studies also showed that BPA concentrations were higher in neonates and young children than in adults.

Some of the studies measuring BPA in human blood were conducted using the enzyme-linked immunosorbent assay. Although this assay is considered less specific than the more precise analytical chemistry methods now favored for measuring BPA, the authors argue these studies are worthy of inclusion in their review because the concentrations they report are in line with what have been detected using the newer methods.

The paper also points out “significant deficiencies” in the two studies that have examined the toxicokinetics of BPA exposure in humans, which they say have been given undue weight in regulatory decision making. The authors note we don’t yet know all the potential sources of exposure to BPA, which makes it impossible to predict toxicokinetics. Because the biomonitoring findings contradict the toxicokinetic studies, the authors recommend in a related commentary in the same issue [*EHP* 118(8):1051–1054; Vandenberg et al.] that biomonitoring data be considered in regulatory decision making whenever available rather than relying only on toxicokinetic models to estimate exposure.

